# Relationship between Serum Lipoprotein Ratios and Insulin Resistance in Polycystic Ovary Syndrome

**DOI:** 10.1155/2012/173281

**Published:** 2012-06-25

**Authors:** Shou-Kui Xiang, Fei Hua, Ying Tang, Xiao-Hong Jiang, Qi Zhuang, Feng-Juan Qian

**Affiliations:** Department of Endocrinology, Third Affiliated Hospital of Suzhou University, Changzhou 213003, China

## Abstract

*Objective*. To investigate the association between serum lipoprotein ratios and insulin resistance in women with polycystic ovarian syndrome (PCOS). *Methods*. 105 PCOS patients and 109 controls were randomly enrolled in the study. Serum levels of luteinizing hormone (LH), follicle-stimulating hormone (FSH), estradiol (E2), total testosterone (T), fasting glucose (FBG), fasting insulin (FINS), serum triglycerides (TG), total cholesterol (TC), high-density lipoprotein (HDL-C), and low-density lipoprotein (LDL-C) levels were checked, and then TG/HDL-C ratio, TC/HDL-C, ratio and LDL-C/HDL-C ratio were calculated. The homeostasis model assessment of insulin resistance (HOMA-IR) was used to calculate the insulin resistance. *Results*. All lipoprotein ratios were significantly higher in PCOS patients as compared to healthy controls (*P* < 0.05). TG/HDL-C ratio, TC/HDL-C ratio, and LDL-C/HDL-C ratio were significantly correlated with HOMA-IR (*P* < 0.05). The ROC curve demonstrated that TC/HDL-C ratio had higher sensitivity and specificity in diagnosing PCOS with insulin resistance. *Conclusion*. This study demonstrates that serum lipoprotein ratio significantly correlates with insulin resistance and can be used as the marker of insulin resistance in PCOS patients.

## 1. Introduction

Polycystic ovary syndrome (PCOS) is the most common endocrine disease and metabolic disorder in adolescence and reproductive women, which is the first reason for female infertility, with the incidence of 5–10% in reproductive women [1]. It is characterized by chronic anovulation, hyperandrogenism, and ovarian polycystic changes under ultrasound in clinic. The etiology of PCOS is still not very clear, but previous studies have shown that PCOS is closely related to lipid metabolism disorder and insulin resistance [2, 3]. 

PCOS is not only reproductive endocrine disease but also metabolic disorder. PCOS patients are often accompanied by obesity, insulin resistance, abnormal glucose metabolism, lipids disorder, hypertension, and other risk factors of cardiovascular disease (CVD) [4]. Lipids disorder is present when TG and LDL-C levels elevate with a lower HDL-C level [5], which is closely related to insulin resistance [6]. 

There is insulin resistance in about 70% of PCOS patients and 10% with diabetes mellitus [7–9]. Insulin resistance is still closely related to CRP, lipids disorder, and other risk factors of CAD even if the glucose metabolism is normal [10]. Using biguanides or thiazolidinediones (TZDs) to treat insulin resistance in PCOS patients has achieved satisfactory clinical effects [11, 12]. It is of great clinical importance to determine whether the PCOS patients are combined with insulin resistance and to take treatments for insulin resistance for symptom improvement and long-term prognosis in PCOS patients.

The majority of those methods to evaluate insulin resistance are complicated to operate, expensive, and time-consuming. There is, therefore, an urgentneedto develop a simple, effective, and economic method to investigate insulin resistance in PCOS. The aim of this research was to study the correlation between serum lipoprotein ratios (TG/HDL-C, TC/HDL-C, and LDL-C/HDL-C) and insulin resistance in PCOS to provide new ideas for evaluation and treatment of PCOS with insulin resistance.

## 2. Materials and Methods

### 2.1. Patients

From February 2010 to October 2011, 105 patients diagnosed with PCOS in the Endocrinology Clinic in our hospital were enrolled in the PCOS group (according to PCOS diagnostic criteria revised in the 2003 Rotterdam meeting). This study was conducted in accordance with the declaration of Helsinki. This study was conducted with approval from the Ethics Committee ofthe Third Affiliated Hospital of Suzhou University. Written informed consent was obtained from all participants. The age of patients ranged from 17 to 36 years (average age: 24.8 years), who had no history of drugs affecting glucose and lipid metabolism. 109 healthy females (with normal menstrual cycle and sex hormone level, no evidence of polycystic ovary on ultrasound examination) were randomly selected as controls (control group) from the Medical Examination Center of our hospital. The age of the controls ranged from 18 to 35 years (an average age: 25.2 years). 

### 2.2. Research Methods

Plasma samples were obtained in early follicular period from all participants after an overnight fasting to measure LH, FSH, E2, T, FBG, FINS, TG, TC, LDL-C, and HDL-C. Then, the ratio of TG/ HDL-C, TC/ HDL-C, and LDL-C/HDL-C was calculated. Their height, weight, waist circumference (WC), and blood pressure were measured, and the body mass index (BMI) was calculated. 

Insulin resistance index was calculated with homeostasis model assessment (HOMA-IR), HOMA-IR = FBG (mmol/L) × FINS (mIU/L)/22.5. The diagnostic criterion of PCOS with insulin resistance (IR) was HOMA-IR > 2.77 [13]. 

### 2.3. Statistical Analysis

Statistical analysis was performed by using SPSS15.0 statistical software. Quantitative data were expressed as mean ± SD. Groups' comparisons were made using independent sample *t*-test. Spearman's correlation analysis was used for correlation analysis. Evaluation of serum lipoprotein ratios with ROC curve was used to diagnose the sensitivity and specificity of PCOS with insulin resistance. *P* values less than 0.05 were considered statistically significant. 

## 3. Results

### 3.1. Clinical and Biochemical Results

There was no difference in age between PCOS and control groups. BMI, WC, SBP, DBP, LH, T, FBG, FINS, HOMA-IR, TG, TC, LDL-C, TG/HDL-C, TC/HDL-C, and LDL-C/HDL-C were significantly higher in PCOS group than in control group. HDL-C was significantly lower in PCOS group than in control group. There was no significant difference between PCOS and control groups in terms of FSH and E2 (Table 1).

### 3.2. Spearman's Correlation Analysis

There was a significant positive correlation between HOMA-IR and TG/HDL-C (*r* = 0.552, *P* < 0.001), TC/HDL-C (*r* = 0.561, *P* < 0.001) and LDL-C/HDL-C (*r* = 0.531, *P* < 0.001), respectively. 

### 3.3. ROC Curve Analysis

**Figure 1:**
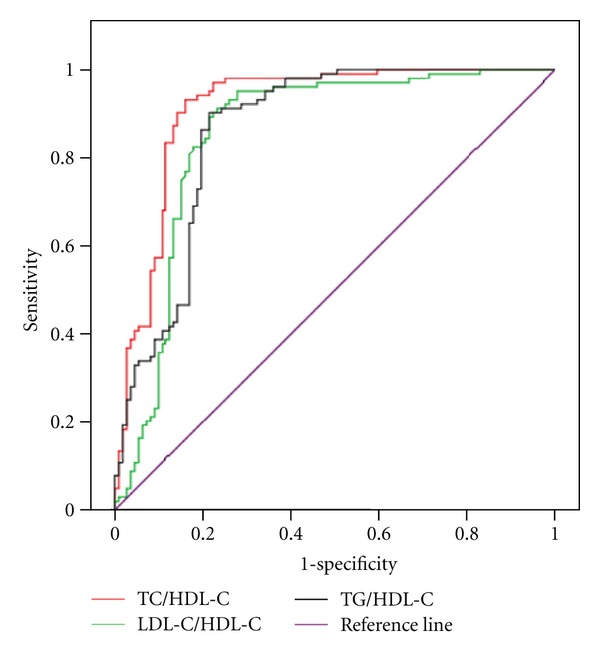
Serum lipoprotein ratios had high sensitivity and specificity for the detection of PCOS with insulin resistance.

It was shown that TG/HDL-C, TC/HDL-C, and LDL-C/HDL-C were effective diagnostic markers for PCOS with insulin resistance, and the area under the ROC curve of TC/HDL-C was the biggest (Table 2, Figure 1) with high sensitivity (93.2%) and specificity (83.8%) (TC/HDL-C > 3.6). 

## 4. Discussion

PCOS is a common endocrine syndrome with a complex etiology and pathogenesis. Recent studies have considered PCOS as not only a reproductive endocrine disease but also metabolic disorder, which is related to hyperinsulinemia, hyperlipidemia, diabetes, and cardiovascular disease. Insulin resistance and concurrent hyperinsulinemia were reported to play a key role in its occurrence and pathophysiology [14, 15]. PCOS symptoms can be improved if insulin resistance is controlled after lifestyle intervention and biguanides and TZDs usage. Thus, a simple, reliable, and economical method to evaluate insulin resistance will greatly contribute to diagnosis, treatment, and prognosis of PCOS. 

Insulin resistance is defined clinically as the inability of a known quantity of exogenous or endogenous insulin to increase glucose uptake and utilization in an individual as much as it does in a normal population. There are many methods of evaluation of insulin resistance, among which the hyperinsulinemic euglycemic clamp technique is considered as the “gold standard” to assess insulin sensitivity; however, it was complicated to operate and time-consuming which, limited its use in clinical and epidemiological studies [16]. Other evaluation methods of insulin resistance, such as the HOMA-IR of minimal model and steady-state model, Bennett index, Li Guangwei index, insulin sensitivity check index (QUICKI), and fasting glucose and insulin ratio (G/I), are more complex, time-consuming, and expensive. Recent studies have shown that 2-hour blood glucose level after oral glucose tolerance test (OGTT) can be used as a reliable indicator for evaluation of insulin resistance in PCOS patients [17], but it is also more complex and expensive. There is, therefore, an urgentneedto develop a simple, effective, and economic method to investigate insulin resistance in PCOS. 

Abnormal lipid metabolism is one of the main metabolic characteristics of PCOS patients. The result of this study showed that PCOS patients had higher TC, LDL-C, and TG and lower HDL-C when compared with age-matched healthy females. These lipid abnormalities were closely related to insulin resistance independent of obesity [18]. Previous studies have shown that serum lipoprotein ratios (TG/HDL-C, TC/HDL-C, and LDL-C/HDL-C) have significant positive correlation with insulin resistance in patients with type 2 diabetes and could be considered as a simple reliable indicator to determine insulin resistance [19]. Similarly, the TG/HDL-C ratio also had significant positive correlation with insulin resistance in nondiabetic obese individuals [20]. In the present study, the HOMA-IR of the PCOS patients was significantly higher than that of the age-matched healthy women, which suggested that insulin resistance had a crucial role in pathogenesis of PCOS. Meanwhile, it was also found that TG/HDL-C, TC/HDL-C, and LDL-C/HDL-C of PCOS patients were significantly higher than those of the age-matched healthy women and had a significant positive correlation with HOMA-IR. ROC curve analysis showed that TG/HDL-C, TC/HDL-C, and LDL-C/HDL-C were effective diagnostic markers of PCOS with insulin resistance, and the area under the ROC curve of TC/HDL-C was the biggest with the highest sensitivity and specificity (TC/HDL-C > 3.6). 

The research showed that serum lipoprotein ratios had significant positive correlation with insulin resistance in PCOS patients, which could be used as a simple, reliable, and economic indicator to evaluate insulin resistance. Thus, it had important clinical significance for diagnosis and treatment options of PCOS.

## Figures and Tables

**Table 1:** Demographic data in PCOS and control groups (^∗^ *P* < 0.05).

Groups	Control group	PCOS group
Age (years)	25.2 ± 4.5	24.8 ± 4.8
BMI (Kg/m^2^)	21.8 ± 3.2	24.2 ± 4.2^∗^
WC (cm)	71.9 ± 6.9	77.2 ± 8.0^∗^
SBP (mmHg)	111 ± 10	123 ± 11^∗^
DBP (mmHg)	73 ± 8	76 ± 8^∗^
FBG (mmol/L)	4.78 ± 0.43	5.57 ± 0.55^∗^
FINS (mIU/L)	8.6 ± 2.6	21.5 ± 5.7^∗^
HOMA-IR	1.83 ± 0.57	5.27 ± 1.04^∗^
TG (mmol/L)	1.43 ± 1.28	2.64 ± 1.04^∗^
TC (mmol/L)	3.69 ± 1.32	5.01 ± 0.69^∗^
LDL-C (mmol/L)	2.10 ± 0.57	2.49 ± 0.29^∗^
HDL-C (mmol/L)	1.27 ± 0.29	1.08 ± 0.16^∗^
TG/HDL-C	1.10 ± 0.99	2.57 ± 1.26^∗^
TC/HDL-C	2.84 ± 1.18	4.71 ± 0.88^∗^
LDL-C/HDL-C	1.61 ± 0.60	2.34 ± 0.50^∗^
LH (mLU/mL)	10.32 ± 2.05	15.69 ± 2.32^∗^
FSH (mLU/mL)	6.06 ± 0.58	6.62 ± 0.75
T (ng/mL)	0.51 ± 0.09	0.97 ± 0.11^∗^
E2 (pg/mL)	64.11 ± 11.45	63.56 ± 12.28

**Table 2:** Serum lipoprotein ratios and the areas under ROC curve for the detection of PCOS with insulin resistance.

Serum lipoprotein ratios	Areas under ROC curve	95% confidence interval	*P *
TG/HDL-C	0.862 ± 0.026	0.811−0.912	<0.001
TC/HDL-C	0.913 ± 0.021	0.873–0.954	<0.001
LDL-C/HDL-C	0.853 ± 0.029	0.797–0.909	<0.001

## References

[1] Adams J, Polson DW, Franks S (1986). Prevalence of polycystic ovaries in women with anovulation and idiopathic hirsutism. *British Medical Journal*.

[2] Galluzzo A, Amato MC, Giordano C (2008). Insulin resistance and polycystic ovary syndrome. *Nutrition, Metabolism and Cardiovascular Diseases*.

[3] Teede HJ, Hutchison S, Zoungas S, Meyer C (2006). Insulin resistance, the metabolic syndrome, diabetes, and cardiovascular disease risk in women with PCOS. *Endocrine*.

[4] Toulis KA, Goulis DG, Mintziori G (2011). Meta-analysis of cardiovascular disease risk markers in women with polycystic ovary syndrome. *Human Reproduction Update*.

[5] Lo JC, Feigenbaum SL, Yang J, Pressman AR, Selby JV, Go AS (2006). Epidemiology and adverse cardiovascular risk profile of diagnosed polycystic ovary syndrome. *Journal of Clinical Endocrinology and Metabolism*.

[6] Valkenburg O, Steegers-Theunissen RPM, Smedts HPM (2008). A more atherogenic serum lipoprotein profile is present in women with polycystic ovary syndrome: a case-control study. *Journal of Clinical Endocrinology and Metabolism*.

[7] Freeman R, Pollack R, Rosenbloom E (2010). Assessing impaired glucose tolerance and insulin resistance in polycystic ovarian syndrome with a muffin test: an alternative to the glucose tolerance test. *Endocrine Practice*.

[8] Farrell K, Antoni MH (2010). Insulin resistance, obesity, inflammation, and depression in polycystic ovary syndrome: biobehavioral mechanisms and interventions. *Fertility and Sterility*.

[9] Ovalle F, Azziz R (2002). Insulin resistance, polycystic ovary syndrome, and type 2 diabetes mellitus. *Fertility and Sterility*.

[10] Karakas SE, Kyoungmi K, Duleba AJ (2010). Determinants of impaired fasting glucose versus glucose intolerance in polycystic ovary syndrome. *Diabetes Care*.

[11] Eisenhardt S, Schwarzmann N, Henschel V (2006). Early effects of metformin in women with polycystic ovary syndrome: a prospective randomized, double-blind, placebo-controlled trial. *Journal of Clinical Endocrinology and Metabolism*.

[12] Dereli D, Dereli T, Bayraktar F, Ozgen AG, Yilmaz C (2005). Endocrine and metabolic effects of rosiglitazone in non-obese women with polycystic ovary disease. *Endocrine Journal*.

[13] Wang Q, Guo T, Tao Y, Song Y, Huang W (2011). Association between serum adipocyte factor level and insulin resistance in polycystic ovarian syndrome. *Gynecological Endocrinology*.

[14] Azziz R (2002). Editorial: polycystic ovary syndrome, insulin resistance, and molecular defects of insulin signaling. *Journal of Clinical Endocrinology and Metabolism*.

[15] Park KH, Kim JY, Ahn CW, Song YD, Lim SK, Lee HC (2001). Polycystic ovarian syndrome (PCOS) and insulin resistance. *International Journal of Gynecology and Obstetrics*.

[16] Traub ML (2011). Assessing and treating insulin resistance in women with polycystic ovarian syndrome. *World Journal of Diabetes*.

[17] Saxena P, Prakash A, Nigam A (2011). Efficacy of 2-hour post glucose insulin levels in predicting insulin resistance in polycystic ovarian syndrome with infertility. *Journal of Human Reproductive Sciences*.

[18] Kalra A, Nair S, Rai L (2006). Association of obesity and insulin resistance with dyslipidemia in Indian women with polycystic ovarian syndrome. *Indian Journal of Medical Sciences*.

[19] Tangvarasittichai S, Poonsub P, Tangvarasittichai O (2010). Association of serum lipoprotein ratios with insulin resistance in type 2 diabetes mellitus. *Indian Journal of Medical Research*.

[20] Brehm A, Pfeiler G, Pacini G, Vierhapper H, Roden M (2004). Relationship between serum lipoprotein ratios and insulin resistance in obesity. *Clinical Chemistry*.

